# Free and bioavailable 25-hydroxyvitamin D thresholds for bone metabolism and their associations with metabolic syndrome in Chinese women of childbearing age

**DOI:** 10.3389/fnut.2023.1131140

**Published:** 2023-08-31

**Authors:** Xiaoyun Shan, Yang Cao, Huidi Zhang, Xiayu Zhao, Siran Li, Yichun Hu, Lichen Yang

**Affiliations:** ^1^Key Laboratory of Trace Element Nutrition of National Health Committee, National Institute for Nutrition and Health, Chinese Center for Disease Control and Prevention, Beijing, China; ^2^Hunan Key Laboratory of Typical Environmental Pollution and Health Hazards, School of Public Health, Hengyang Medical School, University of South China, Hengyang, Hunan, China

**Keywords:** free 25-hydroxyvitamin D, bioavailable 25-hydroxyvitamin D, parathyroid hormone, bone turnover markers, threshold, metabolic syndrome

## Abstract

**Objective:**

The free hormone hypothesis suggests that free and bioavailable 25-hydroxyvitamin D [25(OH)D] may better reflect vitamin D bioactivity. This study aimed to determine the free and bioavailable 25(OH)D characteristics, estimate their thresholds based on parathyroid hormone (PTH) and bone turnover markers (BTMs), assess their associations with the risk of metabolic syndrome (MetS), and evaluate their potential advantages.

**Methods:**

A cross-sectional study was conducted using a nationally representative database (*n* = 1,505, female, 18–45 years). Serum total 25(OH)D, vitamin D-binding protein, albumin, PTH, and BTMs [osteocalcin, β-CrossLaps of type 1 collagen containing cross-linked C-telopeptide (β-CTX), and procollagen type 1 N-terminal propeptide (P1NP)] were measured. Free 25(OH)D and bioavailable 25(OH)D were calculated. The threshold associations of 25(OH)D with PTH and BTMs were analyzed. The relationship between 25(OH)D and MetS risk was examined. An intervention study was then performed in 39 women (18–47 years) to assess the associations of increasing 25(OH)D with PTH and BTMs after vitamin D supplementation.

**Results:**

In the cross-sectional study, the three forms of 25(OH)D were found to have similar distribution characteristics. Free and bioavailable 25(OH)D correlated well with total 25(OH)D. Significant total 25(OH)D cutoffs were observed for PTH (14.19 ng/mL and 18.03 ng/mL), osteocalcin (15.14 ng/mL), β-CTX (14.79 ng/mL), and P1NP (15.08 ng/mL). Free and bioavailable 25(OH)D cutoffs were only found for P1NP (3.47 pg/mL and 1.66 ng/mL, respectively). A total 25(OH)D of <15.14 ng/mL was marginally associated with a higher risk of reduced high-density lipoprotein cholesterol (HDL-C) [odd ratios (OR) = 1.371 (0.991–1.899)]. The ORs of higher versus lower free and bioavailable 25(OH)D levels for reduced HDL-C were 0.770 (0.621–0.956) and 0.772 (0.622–0.958), respectively. The results of the intervention study indicated that PTH and BTMs responded more sensitively to total 25(OH)D than to free or bioavailable 25(OH)D.

**Conclusion:**

Free and bioavailable 25(OH)D only had a threshold effect on P1NP. The active 25(OH)D thresholds could be used for risk assessment of reduced HDL-C. However, no superiority of free or bioavailable 25(OH)D was found based on the response of PTH and BTMs to changes in 25(OH)D in Chinese women of childbearing age following vitamin D supplementation.

**Clinical trial registration:**

http://www.chictr.org.cn, ChiCTR2200058290.

## 1. Introduction

Vitamin D plays a critical role in skeletal health (1) and contributes to extra-skeletal effects, including metabolic syndrome (MetS) (2). The serum total 25(OH)D [Total-25(OH)D, mainly bound to vitamin D-binding protein (VDBP)] level is the best index of nutritional vitamin D status (3). The relationship between Total-25(OH)D and MetS has been investigated in populations worldwide, but the results were inconsistent (4). The free hormone hypothesis (5) suggests that the free and bioavailable forms of 25(OH)D [Free-25(OH)D and Bio-25(OH)D, respectively], which are free or albumin-bound, may better reflect the bioactivity of vitamin D. Because only the free fraction can passively cross the cell membrane, become hydroxylated to the active metabolite [1,25(OH)2D], and exert a biological action (6). In addition, VDBP and Total-25(OH)D are influenced by factors such as liver function, kidney diseases, pregnancy, and genetic background (7, 8), but free vitamin D is independent of these factors and has a better correlation with pathological conditions, especially with liver, kidney, and allergic diseases, and tumor as well as pregnancy. Therefore, it is recommended to determine Free-25(OH)D and/or Bio-25(OH)D under these pathological conditions (9).

Numerous studies have been conducted to confirm the free hormone hypothesis, but confusion remains regarding the superiority of Free-25(OH)D and Bio-25(OH)D in terms of prediction of health outcomes. Some studies have found that Free-25(OH)D and Bio-25(OH)D appear to be superior to Total-25(OH)D as predictive indices of bone health (10–12) or in a similar fashion (13, 14), whereas other have shown that vitamin D status assessed by Total-25(OH)D may better reflect bone health (15, 16). We speculated that these inconsistent conclusions may reflect differences in the ethnicity, sex, and age of the study populations and the methods used to determine Free-25(OH)D and Bio-25(OH)D. Therefore, more observational studies and clinical trials in various physiological and pathological conditions to define optimal Free-25(OH)D and Bio-25(OH)D concentrations are needed.

According to the presently used criteria, vitamin D deficiency is common in Chinese women of childbearing age (17). Studies have identified that there is a threshold association between Total-25(OH)D and serum parathyroid hormone (PTH) (18) and that a lower serum 25(OH)D is associated with some components of MetS (19) in this population. However, there have been no reports on the levels of Free-25(OH)D or Bio-25(OH)D and whether Free-25(OH)D or Bio-25(OH)D can be used to assess vitamin D nutritional status or bioactivity and evaluate the effectiveness of intervention in this population. Therefore, the aim of this research was to investigate whether Free-25(OH)D or Bio-25(OH)D also has a threshold effect on bone metabolism and whether it is more strongly linked to bone metabolism and MetS than Total-25(OH)D in Chinese women of childbearing women.

Two studies were performed. The first was a cross-sectional study based on the 2015 Chinese Chronic Diseases and Nutrition Survey (CCDNS) that was performed to determine the characteristics of Total-25(OH)D, Free-25(OH)D, and Bio-25(OH)D in women of childbearing age and to compare the threshold associations of these three forms of 25(OH)D with PTH and bone turnover markers (BTMs), which are indicators of bone remodeling. If the Free-25(OH)D or Bio-25(OH)D threshold was determined, it would be further used to assess the relationship between vitamin D and MetS risk. The second was an intervention study that was performed to clarify whether the associations of Free-25(OH)D and Bio-25(OH)D with PTH and BTMs were stronger than Total-25(OH)D after vitamin D supplementation.

## 2. Materials and methods

### 2.1. Study design and participants

#### 2.1.1. The cross-sectional study

Women of childbearing age (18–45 years), who were not pregnant or lactating, were recruited for participation from the 2015 CCDNS. The investigation enrolled 1,568 women randomly from 279 survey areas, as described in our previous research (18). Participants with missing anthropometric measurements or other pertinent covariates (*n* = 61) were excluded. A total of 1,505 participants were ultimately included in the cross-sectional study. The study was approved by the Ethical Review Committee of Chinese Center for Disease Control and Prevention (CDC) (No. 201519-B).

#### 2.1.2. The intervention study

Participants were recruited from November to December 2021 in Beijing, China. Women who met the following criteria volunteered for participation: (1) premenopausal women aged 18–50 years (non-pregnant or non-lactating); (2) no medical history of acute or chronic diseases such as hypertension, diabetes, cardiovascular disease, dyslipidemia, kidney disease, cancer, gastrointestinal disorders, and endocrine disorders; and (3) no use of vitamin D, vitamin K, or Ca supplements. Next, serum 25(OH)D concentration was assessed to screen volunteers with inadequate vitamin D status (less than 20 ng/mL). Finally, a total of 40 individuals (18–47 years) were included in the vitamin D supplementation trial. This intervention study was conducted in the Chinese CDC between December 2021 and April 2022.

This study was a 16 weeks, single-group repeated measures design trial. The allocation ratio was 5:3 to high-dose vitamin D3 supplementation (800 IU/d) versus low-dose groups (400 IU/d). A total of 40 participants were randomly assigned to 400 IU/d group (*n* = 15) or 800 IU/d group (*n* = 25). Participants took a commercialized 400 IU or 800 IU vitamin D3 capsule per day (provided by Xiamen Xingsha Pharmaceutical Group Co., Ltd). And no women took birth control pills or other medications that might affect vitamin D absorption and serum VDBP during the intervention period. Finally, 39 participants (97.5%) were available for the 16 weeks follow-up as one person failed to complete the trial. The study has been registered at http://www.chictr.org.cn (ChiCTR2200058290) and was approved by the Ethical Review Committee of Chinese CDC (No. 2018-009).

All procedures performed in the above two studies involving human participants were in accordance with the ethical standards of the committee. Written informed consent was obtained from all participants before their inclusion in the study.

### 2.2. Laboratory assay

Fasting blood samples were obtained between 8:00 and 10:00 AM, and serum samples were stored at −80°C until analysis. The biomarkers were measured following both the manufacturer’s protocol and specialized laboratory assay quality control procedures. Total-25(OH)D, containing 25(OH)D2 and 25(OH)D3, was measured with the use of liquid chromatography-tandem mass spectroscopy (AB Sciex Pte. Ltd., Framingham, MA, United States). The National Institute of Standards and Technology of America (National Institute of Standards and Technology, NIST) standard reference material, SRM 972a, was used to verify the calibration of the assay. The average bias was 2.64% for 25(OH)D2 and 3.13% for 25(OH)D3 compared with Nist SRM 972a. Values of 25(OH)D were expressed in ng/mL (2.5 ng/mL equal to 1 nmol/L). Serum VDBP levels were measured using a polyclonal antibody enzyme-linked immunosorbent assay (ELISA) kit, according to the manufacturer’s instructions (GenWay Biotech, Inc., San Diego, CA, United States). Values of VDBP were expressed in μg/mL (250 μg/mL equal to 4 μmol/L). The PTH, OC, β-CTX, and P1NP were measured by electronic chemiluminescence immunoassay (Roche e601, F Hoffmann-La Roche Ltd., CH4002 Basel, Switzerland). The plasma calcium (Ca) was detected by inductively coupled plasma mass spectrometry (ICP-MS, PerkinElmer, NexION 350, Waltham, MA, United States). Commercially available quality control samples (Clincheck Level-2, Munich, Germany; Seronorm, Level-2, Billingstad, Norway) were used every 10 samples. Serum albumin (Alb), phosphorus (P), creatinine (CRE), high sensitivity C-reactive protein (hsCRP), fasting blood glucose (FBG), high-density lipoprotein cholesterol (HDL-C), total cholesterol, and triglyceride were measured using an automatic biochemical analyzer (Hitachi 7,600, Tokyo, Japan). Alb levels were expressed in g/L (1 g/L equal to 15 μmol/L). All inter- and intra-assay coefficients of variation (CVs) were <4.78%.

Method for selecting and detecting vitamin D metabolism-related single nucleotide polymorphisms (SNPs), including cytochrome P450-2R1 (CYP2R1) rs12794714, VDBP (GC) rs2282679, and vitamin D receptor (VDR) rs2228570 was described in our previous study (19). Then a genetic risk score (GRS) for each subject was obtained by adding the number of A alleles of rs12794714, T alleles of rs2282679, and G alleles of rs2228570 (ranging from 0 to 6).

### 2.3. Data calculation and collection, and potential confounders

Serum Free-25(OH)D and Bio-25(OH)D were calculated using Total-25(OH)D, VDBP, Alb concentrations, and affinity constants for VDBP and Alb, as the previous study reported (10). The mathematical formulas were as follows:

$$\mathrm{Free}-25\left(\mathrm{OH}\right)D=\mathrm{Total}-25\left(\mathrm{OH}\right)D/\left(\begin{array}{l} 1+K_{\mathrm{Alb}}\times \mathrm{Alb}+\\ K_{\mathrm{VDBP}}\times \mathrm{VDBP} \end{array}\right)$$

$$\mathrm{Bio}-25\left(\mathrm{OH}\right)D=\left[\left(K_{\mathrm{Alb}}\times \mathrm{Alb}\right)+1\right]\times \mathrm{Free}-25\left(\mathrm{OH}\right)D.$$

*K*_alb_ is the affinity constant for 25(OH)D and Alb binding (6 × 10^5^/M), and *K*_VDBP_ is the affinity constant for 25(OH)D and VDBP binding (7 × 10^8^/M). Total-25(OH)D, Free-25(OH)D, Bio-25(OH)D, Alb concentration, and VDBP levels are expressed in mol/L.

Height, weight, waist circumferences, systolic blood pressure, and diastolic blood pressure were medically examined. Body mass index (BMI) was calculated as weight kg/height m^2^. As vitamin D status is partly dependent on sunlight exposure, the timing of health examination was divided into three subgroups (no health examination was carried out in summer in this survey): (1) spring (March to May), (2) autumn (September to November), and (3) winter (December to February). Dietary Ca intake was recorded using a Food Frequency Questionnaire (FFQ). Serum Alb-corrected Ca was calculated by the formula [serum Ca – 0.02 × (Alb-40)]. Age, BMI, geographical location, season of blood drawn, lifestyle, dietary Ca intake, time spent outdoors, serum Ca, P, CRE, hsCRP, and GRS were considered as the potential confounders.

### 2.4. Definition of metabolic syndrome

According to the American Diabetes Association (ADA) “Standards of Medical Care in Diabetes-2022” (20) and a joint interim statement (21), MetS was diagnosed when three or more of the following conditions were present: waist circumference >85 cm (women); serum triglyceride concentration >1.7 mmol/L or treated with lipid abnormalities; HDL-C concentration <1.3 mmol/L or treated; blood pressure ≥130/85 mmHg and/or diagnosed and treated with hypertension; or serum FBG concentration ≥5.6 mmol/L or diagnosed and treated with diabetes mellitus.

### 2.5. Statistical analysis

Data analyses were performed using IBM SPSS Statistics 23 and R version 4.0.3 statistical software. Normal distribution was assessed by the Kolmogorov–Smirnov test. If variables were considered non-normally distributed, they were expressed as median and interquartile range (P_25_–P_75_). Non-parametric statistic methods or the Student’s *t*-test were used for the comparison of biochemical parameters between groups. An analytical procedure to predict the Total-25(OH)D, Free-25(OH)D, or Bio-25(OH)D thresholds was performed according to the method reported by Wu et al. (22). First, locally weighted regression and smoothing scatter plots (LOESS) were used to visualize the nonlinear relationship between Total-25(OH)D, Free-25(OH)D, or Bio-25(OH)D and PTH or BTMs, and to obtain potential cutoff values for the three forms of 25(OH)D. Then, nonlinear least squares estimation (NLS) was used to determine the exact value based on the cutoff predicted by LOESS. Finally, the relationship between 25(OH)D and the four endpoints before and after the cutoff value was determined by segmented regression (SR), i.e., slope (β coefficient) and 95% confidence interval (CI), to further confirm the exact cutoff value. Before these procedures, Total-25(OH)D, Free-25(OH)D, or Bio-25(OH)D was adjusted for region, regional type, season, latitude, age, BMI, CRE, hsCRP, Ca intake, time spent outdoors, and GRS by generalized additive model (GAM). Once the thresholds were determined, multivariate logistic analysis was applied to analyze the relationship between the three forms of 25(OH)D and the risk of MetS as well as its components. In the intervention study, scatter plot and linear relationship between change in PTH or BTMs and change in each form of 25(OH)D was analyzed by GraphPad Prism 9. *p* < 0.05 was considered statistically significant.

## 3. Results

### 3.1. General characteristics in the cross-sectional study

In the cross-sectional study, the median age was 30.0 (24.0–37.8) years old. The median BMI was 22.7 (20.3–25.1) kg/m^2^. The median Total-25(OH)D, Free-25(OH)D, and Bio-25(OH)D was 16.6 (12.0–22.6) ng/mL, 3.5 (2.3–5.0) pg/mL and 1.7 (1.1–2.4) ng/mL, respectively. The median PTH, OC, β-CTX, and P1NP concentrations was 34.2 (26.0–44.4) pg/mL, 17.2 (13.8–21.4) ng/mL, 0.4 (0.3–0.5) ng/mL, and 52.8 (40.1–68.4) ng/mL, respectively. The median Alb and VDBP was 53.1 (50.7–55.1) g/L and 390.7 (295.7–450.5) ug/mL, respectively. The median serum corrected Ca was 2.3 (2.2–2.4) mmol/L. The concentration of serum P was 1.4 (1.2–1.5) mmol/L. The prevalence of MetS was 12.6%. The results of renal function indexes and inflammatory markers were shown in Table 1 as well.

**Table 1:** Characteristics of Chinese women of childbearing age (*n* = 1,505, data from the cross-sectional study).

Parameters	Median (P_25_–P_75_) or *N*(%)	Parameters	Median (P_25_–P_75_) or *N*(%)
Region		Total-25(OH)D, ng/mL	16.6 (12.0–22.6)
Eastern	522 (34.7)	Free-25(OH)D, pg/mL	3.5 (2.3–5.0)
Center	478 (31.8)	Bio-25(OH)D, ng/mL	1.7 (1.1–2.4)
Western	505 (33.6)	Alb, g/L	53.1 (50.7–55.1)
Latitude, ^°^N	32.26 (27.8–38.0)	VDBP, ug/mL	390.7 (295.7–450.5)
Urban	891 (59.2)	PTH, pg/mL	34.2 (26.0–44.4)
Season		OC, ng/mL	17.2 (13.8–21.4)
Spring	118 (7.9)	β-CTX, ng/mL	0.4 (0.3–0.5)
Autumn	774 (51.4)	P1NP, ng/mL	52.8 (40.1–68.4)
Winter	613 (40.7)	Corrected Ca, mmol/L	2.3 (2.2–2.4)
Nationality		P, mmol/L	1.4 (1.2–1.5)
Han	1,309 (87.0)	CRE, nmol/L	76.0 (72.0–81.0)
Ethnic minorities	196 (13.0)	hsCRP, mg/L	0.7 (0.2–1.4)
Education		BMI, kg/m^2^	22.7 (20.3–25.1)
Primary	375 (24.9)	Systolic blood pressure, mmHg	115.0 (107.7–123.3)
Medium	876 (58.2)	Diastolic blood pressure, mmHg	71.3 (65.7–76.8)
Advanced	254 (16.9)	Fasting plasma glucose, mmol/L	4.9 (4.6–5.2)
Cigarette smoker	22 (1.5)	Triglyceride, mmol/L	0.9 (0.6–1.2)
Alcohol consumer	322 (21.4)	HDL-C, mmol/L	1.3 (1.1–1.5)
Moderate or high physical activity	228 (15.2)	Total cholesterol, mmol/L	4.2 (3.7–4.8)
Age, years	30.0 (24.0–37.8)	Waist circumference, cm	75.8 (70.0–82.5)
Ca intake, mg/d	286.1 (169.5–430.8)	Elevated triglycerides	186 (12.4)
Time spent outdoors		Reduced HDL-C	716 (47.6)
≤120 min/d	216 (14.4)	Elevated glucose	91 (6.0)
>120 min/d	286 (19.0)	Elevated waist circumferences	492 (32.7)
Unclear	1,003 (66.6)	Elevated blood pressure	102 (6.8)
GRS		MetS	189 (12.6)
0–1	333 (22.1)		
2–3	900 (59.8)		
4–6	272 (18.1)		

25(OH)D, 25-hydroxyvitamin D; Total-25(OH)D, total 25(OH)D; Free-25(OH)D, free 25(OH)D; Bio-25(OH)D, bioavailable 25(OH)D; Alb, albumin; VDBP, vitamin D-binding protein; PTH, parathyroid hormone; OC, osteocalcin; β-CTX, β-CrossLaps of type 1 collagen containing cross-linked C-telopeptide; P1NP, procollagen type 1 N-terminal propeptide; Ca, calcium; P, phosphorus; CRE, creatinine; hsCRP, high sensitivity C-reactive protein; BMI, body mass index; HDL-C, high-density lipoprotein cholesterol; GRS, genetic risk score.

### 3.2. Free-25(OH)D, Bio-25(OH)D, and VDBP concentrations in different subgroups in the cross-sectional study

As shown in Table 2, the distribution characteristics of Free-25(OH)D and Bio-25(OH)D were similar to those of Total-25(OH)D. Free-25(OH)D and Bio-25(OH)D showed regional, latitudinal, regional type, seasonal, and age differences, with relatively higher serum Free-25(OH)D and Bio-25(OH)D levels in eastern regions, low-latitude regions, rural areas, autumn, and higher age groups (*p* < 0.05). Women with outdoor time ≥120 min/d had a higher Bio-25(OH)D concentration (*p* < 0.05). Women in the center and urban area, as well as in the younger age group and Han nationality group had higher VDBP concentrations (*p* < 0.05). Women with reduced HDL-C had lower Total-25(OH)D, Free-25(OH)D, Bio-25(OH)D, and VDBP (*p* < 0.05). In contrast, the three forms of 25(OH)D were higher in women with elevated glucose (*p* < 0.05). No differences of Total-25(OH)D, Free-25(OH)D, Bio-25(OH)D, and VDBP were found between women with and without MetS (*p* > 0.05).

**Table 2:** Total-25(OH)D, Free-25(OH)D, and Bio-25(OH)D and VDBP concentrations of Chinese childbearing women aged 18–45 y (*n* = 1,505, data from the cross-sectional study).

Characteristic	*N* (%)	Total-25(OH)D (ng/mL)	*P*_1_	Free-25(OH)D (pg/mL)	*P*_2_	Bio-25(OH)D (ng/mL)	*P*_3_	VDBP(ug/mL)	*P*_4_
Region			<0.001		0.002		0.007		<0.001
Eastern	522 (34.7)	18.07 (12.94–24.2)#		3.68 (2.47–5.35)a		1.74 (1.17–2.55)a		396.64 (304.24–477.08)	
Center	478 (31.8)	15.84 (11.58–21.75)		3.46 (2.32–4.92)		1.64 (1.12–2.35)		354.34 (291.17–413.81)#	
Western	505 (33.6)	15.86 (11.33–21.19)		3.21 (2.18–4.85)		1.53 (1.06–2.31)		381.2 (295.58–478.76)	
Latitude, ^°^N			<0.001		<0.001		<0.001		0.144
<23.5	141 (9.4)	24.99 (20.96–29.29)#		5.37 (4.02–7.28)#		2.51 (1.82–3.42)#		371.05 (297.04–452.98)	
23.5 ~ 32	602 (40.0)	20.16 (15.57–24.62)#		4.14 (2.96–5.56)#		1.98 (1.41–2.63)#		386.3 (311.84–462.49)	
32 ~ 40.5	524 (34.8)	12.74 (10.11–16.70)		2.69 (1.88–3.87)		1.30 (0.92–1.88)		360.41 (279.16–462.14)	
≥40.5	238 (15.8)	13.20 (9.75–17.49)		2.63 (1.87–3.9)		1.23 (0.91–1.81)		379.67 (296.4–449.28)	
Regional type			<0.001		<0.001		<0.001		0.033
Urban	614 (40.8)	15.24 (11.40–20.88)		3.14 (2.19–4.45)		1.49 (1.05–2.14)		383.13 (322.54–448.63)	
Rural	891 (59.2)	17.61 (12.55–23.87)		3.72 (2.37–5.39)		1.76 (1.15–2.58)		363.66 (280.86–467.18)	
Season			<0.001		0.001		<0.001		0.130
Spring	118 (7.9)	16.16 (11.83–21.83)		3.18 (2.22–4.88)		1.53 (1.02–2.31)		379.42 (309.78–497.45)	
Autumn	774 (51.4)	17.6 (12.79–23.39)b		3.65 (2.51–5.17)b		1.72 (1.2–2.49)#		379.26 (301.98–462.49)	
Winter	613 (40.7)	15.42 (11.17–21.63)		3.28 (2.1–4.84)		1.53 (1.01–2.31)		372.38 (286.41–451.35)	
Age group, y			0.021		<0.001		<0.001		0.005
18 ~ 24	491 (32.6)	15.77 (11.64–20.88)d		3.08 (2.13–4.52)c,d		1.48 (1.02–2.17)c,d		389.58 (315.48–461.15)d	
25 ~ 29	257 (17.1)	16.91 (12.35–23.77)		3.47 (2.43–5.28)		1.69 (1.15–2.52)		364.27 (286.67–458.21)	
30 ~ 34	263 (17.5)	16.26 (11.48–22.00)		3.67 (2.24–4.99)		1.77 (1.11–2.33)		373.7 (294.78–458.48)	
35 ~ 39	213 (14.1)	16.94 (12.29–22.98)		3.47 (2.19–5.06)		1.66 (1.05–2.46)		377.76 (298.47–483.63)	
≥40	281 (18.7)	17.49 (12.45–23.95)		3.84 (2.61–5.69)		1.80 (1.23–2.67)		353.02 (267.85–441.63)	
BMI			0.794		0.996		0.942		0.422
Underweight	136 (9.0)	16.18 (11.94–22.09)		3.48 (2.34–5.07)		1.72 (1.16–2.33)		357.61 (295.19–450.03)	
Normal weight	839 (55.8)	16.75 (12.07–22.97)		3.46 (2.24–5.02)		1.66 (1.09–2.39)		377.8 (298.4–464.36)	
Overweight	377 (25.0)	16.26 (11.98–22.93)		3.39 (2.32–5.09)		1.61 (1.14–2.45)		374.94 (293.98–456.08)	
Obesity	153 (10.2)	16.81 (11.79–20.85)		3.48 (2.31–4.9)		1.67 (1.09–2.31)		379.56 (273.18–461.17)	
Nationality			0.542		0.971		0.871		0.043
Han	1,309 (87.0)	16.63 (12.25–22.29)		3.45 (2.32–4.97)		1.66 (1.12–2.36)		377.76 (299.96–461.19)	
Ethnic minorities	196 (13.0)	16.72 (10.60–24.03)		3.43 (2.16–5.37)		1.59 (1.04–2.53)		348.18 (270.45–456.32)	
Education			0.286		0.972		0.966		0.083
Primary	375 (24.9)	16.46 (11.68–22.27)		3.42 (2.24–5.03)		1.63 (1.06–2.39)		357.78 (282.36–452.55)	
Medium	876 (58.2)	16.88 (12.27–23.04)		3.45 (2.31–5.05)		1.66 (1.12–2.39)		381.37 (296.43–461.95)	
Advanced	254 (16.9)	16.39 (11.99–21.89)		3.48 (2.32–4.91)		1.66 (1.12–2.34)		380.26 (308.16–465.26)	
Time spent outdoors			0.143		0.052		0.046		0.104
≤120 min/d	216 (14.4)	16.96 (12.27–22.3)		3.37 (2.23–4.96)		1.64 (1.13–2.37)		395.49 (300.81–468.34)	
>120 min/d	286 (19.0)	17.89 (12.3–23.67)		3.79 (2.45–5.23)		1.80 (1.18–2.49)#		364.85 (281.46–453.59)	
Unclear	1,003 (66.6)	16.16 (11.86–22.27)		3.37 (2.25–5.00)		1.61 (1.08–2.36)		377.16 (295.22–460.28)	
GRS			<0.001		<0.001		<0.001		0.369
0~1	333 (22.1)	18.28 (12.62–25.08)#		3.83 (2.35–5.55)		1.82 (1.12–2.66)		384.94 (300.27–474.47)	
2~3	900 (59.8)	16.72 (12.02–22.3)#		3.48 (2.34–5.05)		1.67 (1.13–2.38)		373.35 (293.08–457.72)	
4~6	272 (18.1)	14.68 (10.97–20.2)#		3.08 (2.1–4.31)#		1.46 (1.02–2.03)#		374.4 (298.8–461.22)	
MetS			0.343		0.656		0.612		0.050
Yes	189 (12.6)	16.47 (11.80–21.97)		3.47 (2.29–5.18)		1.70 (1.13–2.49)		352.62 (270.70–453.28)	
No	1,316 (87.4)	16.69 (11.99–22.78)		3.45 (2.29–5.01)		1.65 (1.11–2.37)		377.78 (297.96–461.42)	
Elevated triglycerides			0.367		0.904		0.931		0.957
Yes	188 (12.5)	16.39 (11.21–22.99)		3.46 (2.27–4.98)		1.66 (1.12–2.35)		378.70 (287.67–460.76)	
No	1,317 (87.5)	16.65 (12.08–22.44)		3.45 (2.31–5.05)		1.65 (1.11–2.39)		375.94 (295.36–460.76)	
Reduced HDL-C			<0.001		0.013		0.013		0.043
Yes	736 (48.9)	15.81 (11.50–21.77)		3.25 (2.24–4.90)		1.58 (1.10–2.35)		372.78 (286.53–451.93)	
No	769 (51.1)	17.41 (12.55–23.77)		3.61 (2.33–5.16)		1.73 (1.12–2.42)		378.58 (301.16–470.53)	
Elevated glucose			0.014		0.016		0.014		0.265
Yes	91 (6.0)	17.40 (13.56–25.75)		3.90 (2.64–5.95)		1.92 (1.24–2.80)		356.64 (289.86–436.98)	
No	1,414 (94.0)	16.51 (11.88–22.35)		3.44 (2.26–4.98)		1.64 (1.10–2.36)		376.98 (295.43–461.30)	
Elevated waist circumferences			0.189		0.821		0.764		0.197
Yes	492 (32.7)	16.21 (11.86–21.90)		3.46 (2.30–5.00)		1.66 (1.11–2.38)		368.48 (288.21–454.12)	
No	1,013 (67.3)	16.75 (12.05–23.02)		3.45 (2.28–5.05)		1.64 (1.11–2.38)		377.76 (298.40–462.49)	
Elevated blood pressure			0.839		0.236		0.194		0.069
Yes	223 (14.8)	16.50 (12.19–22.34)		3.59 (2.43–5.26)		1.70 (1.17–2.51)		354.66 (280.20–448.03)	
No	1,282 (85.2)	16.69 (11.93–22.55)		3.44 (2.27–4.96)		1.64 (1.10–2.36)		377.84 (296.94–462.56)	
Total	1,505 (100.0)	16.63 (11.96–22.55)		3.45 (2.29–5.03)		1.65 (1.11–2.38)		376.36 (295.36–460.72)	

25(OH)D, 25-hydroxyvitamin D; Total-25(OH)D, total 25(OH)D; Free-25(OH)D, free 25(OH)D; Bio-25(OH)D, bioavailable 25(OH)D; VDBP, vitamin D-binding protein; BMI, body mass index; GRS, genetic risk score; MetS, metabolic syndrome. #, compared with other groups; a, Eastern vs. Western; b, Autumn vs. Winter; c, 18~24 y vs. 25~30 y; d, 18~24 y vs. ≥40 y; *p* < 0.05.

### 3.3. Correlation between Free-25(OH)D or Bio-25(OH)D and Total-25(OH)D in the cross-sectional study

**Figure 1:**
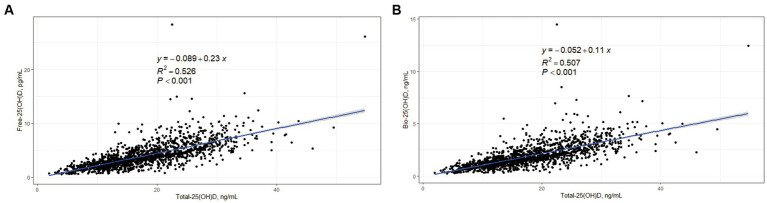
Correlations between Free-25(OH)D, Bio-25(OH)D, and Total-25(OH)D in women of childbearing age (data from the cross-sectional study). **(A)** Correlation between Free-25(OH)D and Total-25(OH)D; **(B)** Correlation between Bio-25(OH)D and Total-25(OH)D.

In the cross-sectional study, Free-25(OH)D and Bio-25(OH)D were positively associated with Total-25(OH)D (*p* < 0.001), with an estimated *R*^2^ value of 0.526 and 0.507, respectively (Figure 1).

### 3.4. Threshold-associations of Free-25(OH)D and Bio-25(OH)D with PTH and BTMs in the cross-sectional study

**Figure 2:**
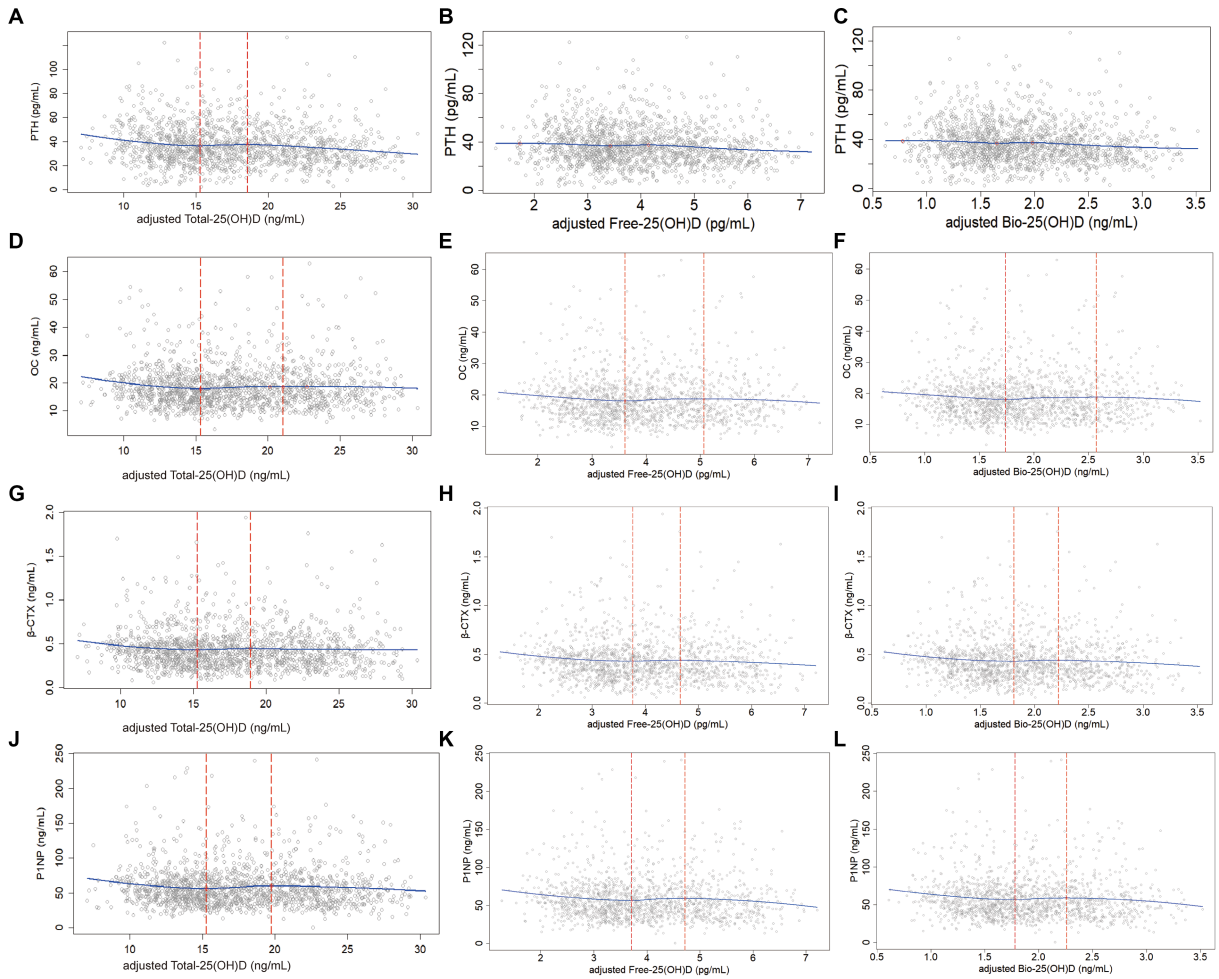
Non-linear associations (LOESS) between adjusted Total-25(OH)D, Free-25(OH)D or Bio-25(OH)D and PTH as well BTMs (data from the cross-sectional study): y-PTH, OC, β-CTX, or P1NP; x-adjusted Total-25(OH)D, Free-25(OH)D, or Bio-25(OH)D; **(A)**, **(D)**, **(G)**, and **(J)**: Non-linear associations of Total-25(OH)D with PTH as well as BTMs; **(B)**, **(E)**, **(H)**, and **(K)**: Non-linear associations of Free-25(OH)D with PTH as well as BTMs; **(C)**, **(F)**, **(I)**, and **(L)**: Non-linear associations of Bio-25(OH)D with PTH as well as BTMs. In the analysis for PTH, one sample was removed because the PTH concentration exceeded 150 pg/mL.

In the predicted models, Total-25(OH)D, Free-25(OH)D, and Bio-25(OH)D were adjusted for region, regional type, season, latitude, age, BMI, CRE, hsCRP, Ca intake, time spent outdoors, and GRS. After adjustment, the LOESS scatter plots showed the non-linear associations of Total-25(OH)D with PTH or BTMs and their potential cutoffs, but such association was not found between Free-25(OH)D or Bio-25(OH)D and PTH (Figure 2).

Table 3 shows the exact 25(OH)D thresholds predicted by NLS and SR. The first adjusted cutoff of Total-25(OH)D for PTH, OC, β-CTX, and P1NP was 14.19 (12.23, 16.14) ng/mL, 15.14 (13.56, 16.71) ng/mL, 14.79 (12.11, 17.47) ng/mL, and 15.08 (12.89, 17.27) ng/mL, respectively. Below these cutoffs, Total-25(OH)D was negatively associated with PTH, OC, β-CTX, and P1NP (*p* < 0.05). Above these cutoffs, OC, β-CTX, and P1NP began to level off, while PTH declined again after the second cutoff [18.03 (15.11, 20.96) ng/mL]. Free-25(OH)D and Bio-25(OH)D cutoffs with their 95% CIs [cutoff_Free-25(OH)D-1_ = 3.47 (2.87, 4.08) pg/mL; cutoff_Bio-25(OH)D-1_ = 1.66 (1.37, 1.96) ng/mL] estimated by the NLS and SR were only significant for P1NP. Below the first cutoff, P1NP was negatively associated with Free-25(OH)D and Bio-25(OH)D levels [β_Free-25(OH)D-1_ = −6.14 (−11.09, −1.20), *p* < 0.05; β_Bio-25(OH)D-1_ = −12.97 (−23.28, −2.65), *p* < 0.05], and then entered a plateaus. Similarly, PTH showed a decreasing trend after the second Free-25(OH)D and Bio-25(OH)D cutoff in the models.

**Table 3:** Associations between serum Total-25(OH)D, Free-25(OH)D, and Bio-25(OH)D level and PTH or BTMs below and above the adjusted cutoffs of 25(OH)D, estimated by nonlinear least squares estimation and segmented regression (data from the cross-sectional study).

	Below cutoff_1_ *β* (95%CI)	Cutoff_1_	Above cutoff_1_ *β* (95%CI)	Cutoff_2_	Above cutoff_2_ *β* (95%CI)
Total-25(OH)D					
PTH	−1.35 (−2.35,-0.34)	14.19 (12.23,16.14)	0.63 (−0.74,2.00)	18.03 (15.11,20.96)	−0.69 (−1.13,-0.25)
OC	−0.55 (−0.91,-0.19)	15.14 (13.56,16.71)	1.03 (−2.18,4.25)	16.39 (13.91,18.86)	−0.001 (−0.16,0.15)
β-CTX	−0.01 (−0.02,-0.00003)	14.79 (12.11,17.47)	0.01 (−0.03,0.05)	17.22 (11.83,22.62)	−0.002 (−0.007,0.004)
P1NP	−1.62 (−3.03,-0.21)	15.08 (12.89,17.27)	1.208 (−0.52,2.94)	20.08 (16.62,23.54)	−0.83 (−1.92,0.26)
Free-25(OH)D					
PTH	−2.29 (−4.87, 0.29)	3.61 (3.16, 4.05)	10.65 (−26.27, 47.57)	3.85 (3.42, 4.29)	−2.57 (−4.17, −0.98)
OC	−1.23 (−2.65, 0.19)	3.38 (2.46, 4.29)	0.21 (−0.33, 0.75)		
β-CTX	−0.04 (−0.08, 0.002)	3.42 (2.52,4.31)	0.01 (−0.04, 0.06)	4.74 (3.24,6.24)	−0.02 (−0.07, 0.02)
P1NP	−6.14 (−11.09, −1.20)	3.47 (2.87,4.08)	4.08 (−3.73, 11.89)	4.65 (3.75,5.55)	−4.00 (−8.82, 0.83)
Bio-25(OH)D					
PTH	15.70 (−18.90, 50.29)	1.09 (0.78, 1.40)	−7.19 (−22.13, 7.74)	1.51 (0.74, 2.29)	−2.38 (−4.53, −0.23)
OC	−2.68 (−5.72, 0.36)	1.60 (1.19, 2.01)	0.51 (−0.60, 1.61)		
β-CTX	−0.08 (−0.16, 0.001)	1.66 (1.26, 2.06)	0.04 (−0.09, 0.17)	2.21 (1.58, 2.83)	−0.05 (−0.13, 0.03)
P1NP	−12.97 (−23.28, −2.65)	1.66 (1.37, 1.96)	8.16 (−8.36, 24.67)	2.21 (1.74, 2.67)	−7.06 (−16.86, 2.73)

Total-25(OH)D, Free-25(OH)D, and Bio-25(OH)D were adjusted for region, regional type, season, latitude, age, BMI, CRE, hsCRP, Ca intake, time spent outdoors, and GRS. Total-25(OH)D, total 25(OH)D; Free-25(OH)D, free 25(OH)D; Bio-25(OH)D, bioavailable 25(OH)D; PTH, parathyroid hormone; OC, osteocalcin; β-CTX, β-CrossLaps of type 1 collagen containing cross-linked C-telopeptide; P1NP, procollagen type 1 N-terminal propeptide; GRS, genetic risk score. In the analysis of the 25(OH)D cutoff for PTH, one sample was removed because the PTH concentration exceeded 150 pg/mL.

### 3.5. Risk of MetS and its components in subgroups stratified according to the thresholds determined in the cross-sectional study

In the cross-sectional study, women were divided into 3 subgroups based on the two predicted Total-25(OH)D cutoffs (15.14 and 18.03 ng/mL). The women were also stratified into two subgroups according to the threshold of Free-25(OH)D (3.47 pg/mL) or Bio-25(OH)D (1.66 ng/mL). In the analysis of model 2, with adjustments for age, race, education, physical activity, smoking, drinking, and BMI, and other components of MetS, women with Total-25(OH)D < 15.14 ng/mL had marginal higher risk of reduced HDL-C than those with Total-25(OH)D between 15.14 ng/mL and 18.03 ng/mL (OR: 1.371, 95% CI: 0.991–1.899, *p* = 0.056). And higher Free- or Bio-25(OH)D had a lower risk of reduced HDL-C (OR_Free-25(OH)D_: 0.770, 95% CI: 0.621–0.956; OR_Bio-25(OH)D_: 0.772, 95% CI: 0.622–0.958) than the lower group. But Bio-25(OH)D, Free-25(OH)D, and Bio-25(OH)D levels were not found significantly associated with the prevalence of MetS, elevated triglycerides, elevated waist circumferences, or elevated blood pressure. Although an increase in serum Bio-25(OH)D, Free-25(OH)D, and Bio-25(OH)D was significantly associated with an increased risk of elevated glucose, no significant associations were found between elevated glucose and Total-25(OH)D, Free-25(OH)D, or Bio-25(OH)D levels after stratification (Table 4).

**Table 4:** Multivariate logistic regression for the association of MetS and individual components of MetS with vitamin D status (data from the cross-sectional study).

	Total-25(OH)D[OR (95%CI)]				Free-25(OH)D[OR (95%CI)]			Bio-25(OH)D[OR (95%CI)]		
	Continuous	<15.14 ng/mL	15.14–18.03 ng/mL	≥18.03 ng/mL	Continuous	<3.47 pg/mL	≥3.47 pg/mL	Continuous	<1.66 ng/mL	≥1.66 ng/mL
MetS										
Model 1	0.986 (0.962–1.01)	1.031 (0.612–1.736)	reference	0.811 (0.479–1.372)	0.978 (0.903–1.058)	reference	0.857 (0.602–1.221)	0.963 (0.819–1.131)	reference	0.908 (0.638–1.292)
Elevated triglycerides										
Model 1	0.993 (0.972–1.015)	1.667 (0.987–2.814)	reference	1.416 (0.836–2.398)	0.969 (0.902–1.042)	reference	0.983 (0.718–1.347)	0.952 (0.823–1.102)	reference	0.976 (0.713–1.337)
Model 2	1.002 (0.98–1.025)	1.536 (0.892–2.645)	reference	1.531 (0.887–2.643)	0.979 (0.909–1.054)	reference	1.066 (0.768–1.479)	0.971 (0.836–1.128)	reference	1.051 (0.758–1.458)
Reduced HDL-C										
Model 1	0.969 (0.955–0.984)	1.444 (1.049–1.987)	reference	0.975 (0.709–1.342)	0.962 (0.918–1.008)	reference	0.78 (0.631–0.964)	0.924 (0.84–1.016)	reference	0.784 (0.634–0.968)
Model 2	0.969 (0.954–0.983)	1.371 (0.991–1.899)	reference	0.933 (0.675–1.291)	0.963 (0.919–1.01)	reference	0.77 (0.621–0.956)	0.925 (0.839–1.019)	reference	0.772 (0.622–0.958)
Elevated glucose										
Model 1	1.046 (1.018–1.075)	0.659 (0.347–1.251)	reference	0.951 (0.518–1.745)	1.122 (1.039–1.211)	reference	1.311 (0.845–2.033)	1.263 (1.085–1.47)	reference	1.45 (0.933–2.253)
Model 2	1.048 (1.019–1.077)	0.646 (0.339–1.231)	reference	0.954 (0.519–1.753)	1.123 (1.041–1.212)	reference	1.323 (0.852–2.055)	1.265 (1.087–1.473)	reference	1.463 (0.94–2.277)
Elevated waist circumferences										
Model 1	0.984 (0.964–1.005)	1.609 (0.988–2.622)	reference	1.212 (0.742–1.981)	0.972 (0.907–1.043)	reference	1.062 (0.776–1.452)	0.947 (0.819–1.095)	reference	1.13 (0.826–1.545)
Model 2	0.986 (0.966–1.007)	1.601 (0.97–2.642)	reference	1.245 (0.753–2.059)	0.978 (0.91–1.05)	reference	1.102 (0.801–1.517)	0.956 (0.824–1.109)	reference	1.173 (0.853–1.615)
Elevated blood pressure										
Model 1	1.006 (0.986–1.026)	0.86 (0.55–1.344)	reference	0.792 (0.507–1.237)	1.01 (0.946–1.079)	reference	1.026 (0.757–1.39)	1.033 (0.905–1.18)	reference	1.006 (0.743–1.363)
Model 2	1.006 (0.986–1.027)	0.794 (0.504–1.251)	reference	0.743 (0.473–1.168)	1.015 (0.949–1.086)	reference	1.008 (0.74–1.374)	1.042 (0.909–1.195)	reference	0.989 (0.726–1.348)

Model 1 was adjusted for age, race, education, physical activity, smoking, drinking, and BMI. Model 2 was adjusted for age, race, education, physical activity, smoking, drinking, BMI, plus additionally adjusted for other components of the MetS. Total-25(OH)D, Total 25(OH)D; Free-25(OH)D, free 25(OH)D; Bio-25(OH)D, bioavailable 25(OH)D; HDL-C, high-density lipoprotein cholesterol.

### 3.6. General characteristics in the intervention study

In the intervention study, the average age was 31.2 ± 7.5 years and the average BMI was 21.5 ± 2.5 kg/m^2^. Age and BMI were not significantly different between 400 IU/d and 800 IU/d groups. Baseline Total-25(OH)D (9.9 ± 3.0 ng/mL vs. 11.7 ± 4.0 ng/mL; *p* = 0.154), Free-25(OH)D (2.2 ± 0.7 pg/mL vs. 2.4 ± 0.9 pg/mL, *p* = 0.602), and Bio-25(OH)D (1.0 ± 0.3 ng/mL vs. 1.1 ± 0.4 ng/mL, *p* = 0.611) showed no significant differences between the 400 IU/d and 800 IU/d group (data not shown).

### 3.7. Total-25(OH)D, Free-25(OH)D, or Bio −25(OH)D associations with PTH and BTMs after vitamin D supplementation

**Figure 3:**
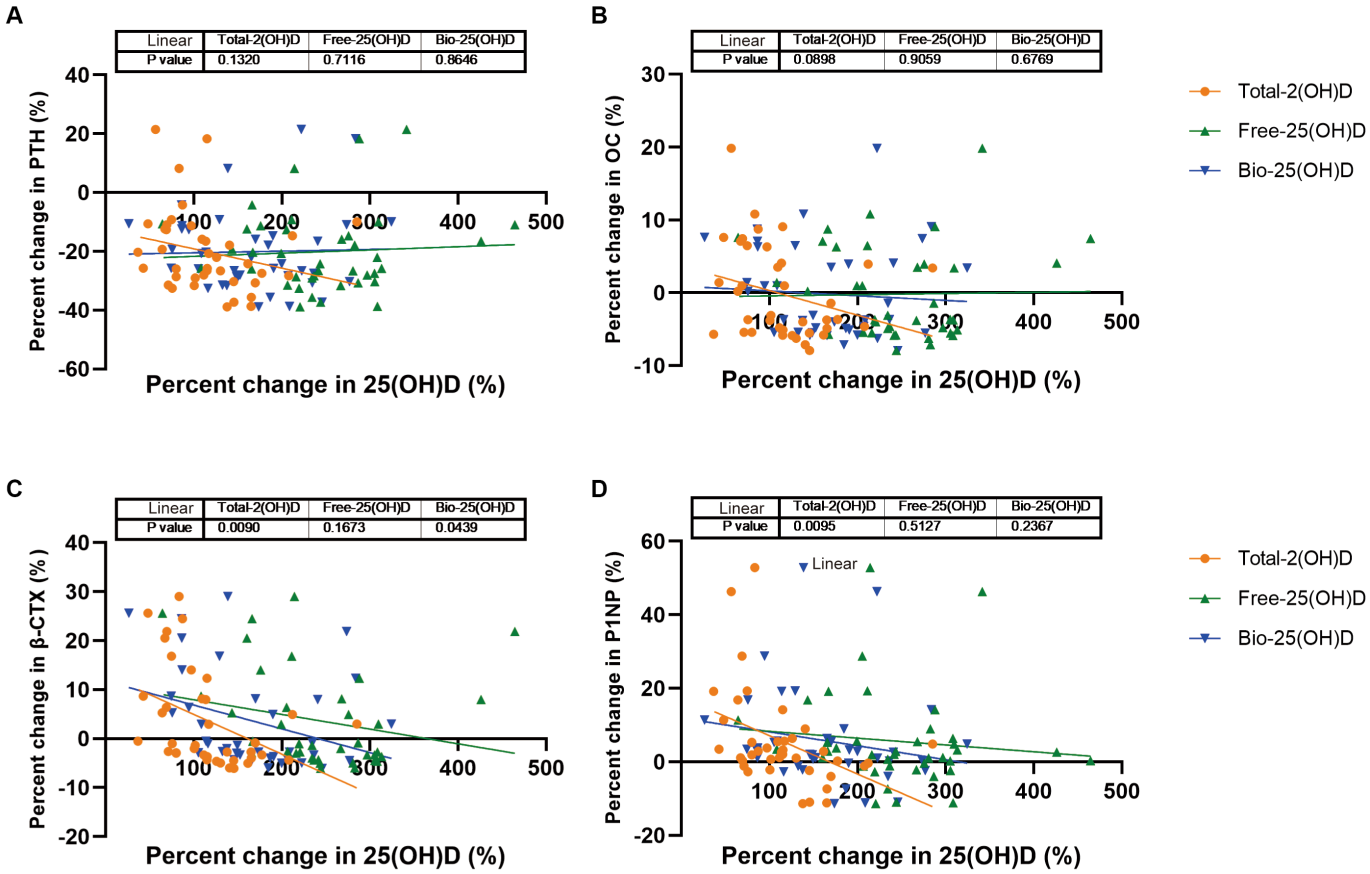
PTH and BTMs response to change in Total-25(OH)D vs. Free-25(OH)D or Bio-25(OH)D following vitamin D supplementation (data from the intervention study). **(A)** PTH response to change in Total-25(OH)D vs. Free-25(OH)D or Bio-25(OH)D; **(B)** OC response to change in Total-25(OH)D vs. Free-25(OH)D or Bio-25(OH)D; **(C)** β-CTX response to change in Total-25(OH)D vs. Free-25(OH)D or Bio-25(OH)D; **(D)** P1NP response to change in Total-25(OH)D vs. Free-25(OH)D or Bio-25(OH)D.

In the intervention study, after vitamin D supplementation, the three forms of 25(OH)D increased in both groups, with no group differences. As Free-25(OH)D turned to be more responsive to vitamin D supplementation than Total-25(OH)D in week 4 (Supplementary Figure S1), we further examined the associations between percent change in PTH (or BTMs) and percent change in Total-25(OH)D vs. Free-25(OH)D or Bio-25(OH)D for the 39 participants, after 4 weeks of supplementation. As shown in Figure 3, percent change in β-CTX and P1NP was significantly associated with percent change in Total-25(OH)D after adjustment for vitamin D supplementation regimen, baseline 25(OH)D, age, and BMI. Meanwhile, percent change in β-CTX was significantly correlated with percent change in Bio-25(OH)D. However, no association was found between percent change in any of these endpoints with percent change in Free-25(OH)D after adjustment for the same covariates.

## 4. Discussion

This study is the first to use a nationally representative database to determine the characteristics of serum Free-25(OH)D and Bio-25(OH)D and to analyze their threshold associations with PTH and BTMs in Chinese women of childbearing age. The thresholds of Free-25(OH)D (3.47 pg/mL) and Bio-25(OH)D (1.66 ng/mL) found in terms of P1NP were further used for assessment of the MetS risk in this population. Lower Free-25(OH)D and Bio-25(OH)D levels were associated with reduced HDL-C. However, as markers of vitamin D metabolism, Free-25(OH)D and Bio-25(OH)D did not show superiority over Total-25(OH)D in this study.

Several studies have evaluated the levels of Free-25(OH)D or Bio-25(OH)D in Chinese adults (23), postmenopausal women (24), and patients (25, 26). The median Total-25(OH)D (16.6 ng/mL) and median Bio-25(OH)D (1.65 ng/mL) levels in our cross-sectional study were slightly higher than the adults (aged 20–45 years) levels [mean Total-25(OH)D, 13.12 ng/mL; mean Bio-25(OH)D, 1.08 ng/mL] measured in winter in Shanghai, China, which is at a latitude between 30°40′ N and 31°53′ N (23). We suspect that this difference may be due to the latitude and season during which sampling was performed because a large proportion of our participants were from low-latitude regions or sampled in autumn, resulting in longer ultraviolet B exposure. As shown in our study, participants at lower latitudes or sampled in autumn had higher Total-25(OH)D, Free-25(OH)D, and Bio-25(OH)D concentrations. An epidemiological survey showed that the mean serum 25(OH)D level increased slightly with age (from 18 to 65 years) (27), which is consistent with our finding that younger women had lower Total-25(OH)D, Free-25(OH)D, and Bio-25(OH)D concentrations. Moreover, the Bio-25(OH)D level in this study was lower than that in postmenopausal women [2.91 (2.11–4.17) ng/mL], sampled in Shanghai (24). Furthermore, we found that Free-25(OH)D or Bio-25(OH)D showed characteristics similar to those of Total-25(OH)D in terms of longitudinal, urban–rural, and genetic profiles.

It has been confirmed that Free-25(OH)D is strongly correlated with Total-25(OH)D in most normal populations whether measured directly or indirectly (28). Similarly, in our study, Free-25(OH)D and Bio-25(OH)D were positively correlated with Total-25(OH)D. Previous studies have compared the associations of Free-/Bio-25(OH)D and Total-25(OH)D with various markers of vitamin D bioactivity, including serum Ca (29), PTH (30), Ca absorption, BTMs (31), bone mineral density (11, 15), and endogenous antimicrobial peptides (32). Some but not all of these comparisons suggest that Free-25(OH)D is a better biomarker of the effects of vitamin D. For example, a review from Bikle et al. concluded that measurement of the free level might provide a better index of vitamin D status than the total level in some clinical situations (6). However, Michaelsson et al. concluded that vitamin D status assessed by direct measurement of Free-25(OH)D was not a better indicator of bone mineral density than Total-25(OH)D (15). Therefore, the free hormone hypothesis regarding 25(OH)D remains unproven.

Based on the association of vitamin D with bone health, the optimal threshold of Total-25(OH)D is mainly determined by skeletal outcomes, such as bone mineral density (22, 33), PTH (34), and BTMs (35) because serum Total-25(OH)D has been shown to be positively/negatively associated these parameters at lower but not at higher Total-25(OH)D levels. Our previous study in Chinese women of childbearing age explored the threshold of vitamin D sufficiency (15.25–16.75 ng/mL) in terms of Total-25(OH)D, during which serum PTH plateaued (18). However, no consensus on the optimal serum Total-25(OH)D concentration for the PTH platform and/or maximal suppression has emerged from the various studies (34), with some studies finding no such threshold or plateau (36). Some studies have explored the Free-25(OH)D and Bio-25(OH)D threshold for vitamin D deficiency (23, 37). For example, Celik et al. estimated the cutoff values for all forms of vitamin D in terms of vitamin D deficiency based on PTH in obese and healthy adolescents using local polynomial regression models. The Free-25(OH)D and Bio-25(OH)D threshold for the entire study population were 13 pg/mL and 5.4 ng/mL, respectively (37). Yao et al. also reported a PTH-based Bio-25(OH)D threshold of 5.8 nmol/L (2.32 ng/mL) in Chinese adults in Shanghai using LOESS (23). However, no Free-25(OH)D or Bio-25(OH)D threshold for PTH was found in our study. Despite the limited research available, we have identified differences in the threshold association of Free-25(OH)D or Bio-25(OH)D with PTH in different populations. Therefore, more studies are needed to identify appropriate Free-25(OH)D and Bio-25(OH)D concentrations in specific populations.

Vitamin D has direct effects on bone cells, including simultaneous activation of both osteoblasts and osteoclasts, since the bone cells express VDR. But it is somewhat unclear whether the result is bone formation, bone resorption, or a neutral effect (38). PTH treatment also concomitantly stimulates new bone formation and bone resorption (39). It was reported that 25(OH)D levels in healthy premenopausal women were positively related with serum CTX and negatively with serum PTH and P1NP (40, 41). Cross-sectional studies also showed that the nonlinear relationship between vitamin D and PTH and BTMs was not entirely consistent (35, 42). We then analyzed the nonlinear relationships between the active forms of 25(OH)D and BTMs in this study population, and compared the results with those for Total-25(OH)D. The 25(OH)D level has been shown to be associated with seasonality, latitude (43), age, BMI (44), serum Ca, P, and CRE levels (45), inflammatory markers (46), genetic factors (47), and other factors. Thus, we further controlled the influence of time spent outdoors, serum P, CRE, and hsCRP levels, and gene polymorphisms based on the preliminary analysis (18). In the present study, the adjusted Total-25(OH)D thresholds were observed for each BTM by LOESS, NLS, and SR. The Total-25(OH)D thresholds obtained for BTMs were relatively concentrated, ranging from 14.79 ng/mL to 15.14 ng/mL. However, the Free-25(OH)D and Bio-25(OH)D thresholds were only obtained for P1NP. An alternative explanation for this finding is that the response of different BTMs to changes in active forms of vitamin D may be inconsistent among women of childbearing age with relatively healthy bones. NLS and SR suggested that the significant Free-25(OH)D and Bio-25(OH)D cutoff were 3.47 pg/mL and 1.66 ng/mL, respectively. The Bio-25(OH)D cutoff was lower than the value of 5.8 nmol/L (2.32 ng/mL) in another Chinese study performed in adults of both sexes, which showed that PTH reached a virtual plateau at this cutoff value (23). This finding might reflect differences in study populations and endpoints. Of course, these results suggest that Free-25(OH)D and Bio-25(OH)D might also be indicators that can be used to explore vitamin D deficiency in terms of bone remodeling in women of childbearing age. Nevertheless, more studies are warranted to verify these findings.

Many studies have reported that vitamin D levels are significantly associated with blood lipid levels. Liu et al. found that deficiency of vitamin D [serum 25(OH)D < 20 ng/mL] might be a risk factor for elevated TG and reduced HDL-C after adjustment for other components in elderly Chinese individuals (48). Similarly, our previous analysis also found that the serum 25(OH)D levels showed a significant association with elevated TG and reduced HDL-C (19). Possible mechanisms include the following. First, vitamin D and cholesterol share a common metabolic substrate, namely 7-dehydrocholesterol (49). Second, active 25(OH)D metabolites can regulate mitochondrial activity, lipid metabolism, and adipogenesis via VDR signaling (50). In this study, we also found that the three forms of 25(OH)D were lower in women with reduced HDL-C, while no differences were found between women with and without MetS. Lower Total-25(OH)D, Free-25(OH)D, and Bio-25(OH)D, which were stratified according to thresholds determined by NLS and SR, were associated with reduced HDL-C. The potential mechanism underlying the association between Free-25(OH)D and Bio-25(OH)D and HDL-C may be the same as Total-25(OH)D. Although an increase in serum Free-25(OH)D of 1 pg/mL and an increase in Total/Bio-25(OH)D of 1 ng/mL were significantly associated with an increased risk of elevated glucose, no associations were found after stratification. Free-25(OH)D, Bio-25(OH)D, and Total-25(OH)D thresholds act in the same way with regard to assessment of the risk of MetS. These findings also suggested that Free-/Bio-25(OH)D did not offer additional advantages over Total-25(OH)D regarding its association with metabolic traits, similar to a Mexican study (51).

Furthermore, we performed a 16 weeks intervention study to clarify whether Free-25(OH)D and Bio-25(OH)D would respond more sensitively to vitamin D supplementation in vitamin D-deficient women and whether the associations of Free-25(OH)D and Bio-25(OH)D with PTH and BTMs would become stronger in response to an increase in Free-25(OH)D and Bio-25(OH)D. The results within 4 weeks suggested that suppression of BTMs may be more strongly driven by Total-25(OH)D, which is consistent with the findings of another intervention study in participants aged >65 years, namely, that Total-25(OH)D but not Free-25(OH)D or Bio-25(OH)D (calculated) had a positive relationship with percent change in bone mineral density at the femoral neck after 12 months of vitamin D supplementation (52). However, the results of such studies have been inconsistent. A study by Smith et al. showed that there was a progressive decrease in serum PTH with increasing doses of vitamin D and that the percentage change in PTH was similar for Free-25(OH)D (directly measured) and Total-25(OH)D (53). Another study in adults aged ≥18 years with baseline 25(OH)D levels <20 ng/mL showed that an increase in Free-25(OH)D but not in Total-25(OH)D was significantly associated with a decrease in PTH early in the repletion course (baseline to 4 weeks) when 25(OH)D levels increase most rapidly (54). These inconsistencies may reflect differences in study populations and in the methodology used to determine Free-25(OH)D and Bio-25(OH)D levels. Therefore, further studies in specific populations using standardized and consistent measuring methods for Free-25(OH)D and Bio-25(OH)D are warranted.

This research has some limitations. First, the Free-25(OH)D and Bio-25(OH)D levels may have been overestimated because they were calculated based on the Total-25(OH)D, Alb, and VDBP levels in serum. Future comparisons should include direct measurement of Free-25(OH)D and Bio-25(OH)D. Second, although we considered the influence of season and time spent outdoors on the results, we lack detailed data on dietary habits, sunlight exposure, and use of sunscreen preparations.

## 5. Conclusion

In this research, which included a cross-sectional study and another with an interventional design, we investigated Free-25(OH)D and Bio-25(OH)D thresholds based on PTH and three BTMs with adjustment for environmental and genetic confounders, assessed the risk of MetS according to these thresholds, and compared the possible advantages of using Free-25(OH)D and Bio-25(OH)D over Total-25(OH)D. Unlike Total-25(OH)D, only the P1NP platform was found for both active forms of 25(OH)D. Although the thresholds determined for all three forms of 25(OH)D could also predict the risk of reduced HDL-C, no superiority of Free-25(OH)D or Bio-25(OH)D was found based on the PTH and BTMs response to changes in 25(OH)D in Chinese women of childbearing age during the intervention study. However, more cohort and intervention studies are needed to confirm our findings.

## Data availability statement

The original contributions presented in the study are included in the article/Supplementary material, further inquiries can be directed to the corresponding author.

## Ethics statement

The studies involving humans were approved by the Ethical Review Committee of Center for Disease Control and Prevention (CDC) (No. 2018-009 and No. 201519-B). The studies were conducted in accordance with the local legislation and institutional requirements. The participants provided their written informed consent to participate in this study.

## Author contributions

XS: data curation, formal analysis, methodology, software, visualization, writing – original draft, and writing – review and editing. YC, HZ, XZ, and SL: resources, methodology, and software. YH: investigation and conceptualization. LY: conceptualization, funding acquisition, project administration, and supervision. All authors contributed to the article and approved the submitted version.

## Funding

This work was supported by the National Natural Science Foundation of China (Grant number 81872627).

## Conflict of interest

The authors declare that the research was conducted in the absence of any commercial or financial relationships that could be construed as a potential conflict of interest.

## Publisher’s note

All claims expressed in this article are solely those of the authors and do not necessarily represent those of their affiliated organizations, or those of the publisher, the editors and the reviewers. Any product that may be evaluated in this article, or claim that may be made by its manufacturer, is not guaranteed or endorsed by the publisher.
