# Supplementary material for: Free and bioavailable 25-hydroxyvitamin D thresholds for bone metabolism and their associations with metabolic syndrome in Chinese women of childbearing age
Source: Front Nutr. 2023 Aug 31;10:1131140. doi: 10.3389/fnut.2023.1131140 (PMC10509557; doi:10.3389/fnut.2023.1131140)
Supplement: Supplementary file 1 [file Data_Sheet_1.PDF]

![frontiers]

Supplementary Material

Free and bioavailable 25-hydroxyvitamin D thresholds for bone metabolism and their associations with metabolic syndrome in Chinese women of childbearing age

Xiaoyun Shan[1,2], Yang Cao[2], Huidi Zhang[2], Xiayu Zhao[2], Siran Li[2], Yichun Hu[2], Lichen Yang[2*]

*** Correspondence:** Lichen Yang, yanglc@ninh.chinacdc.cn

1 Supplementary Figures and Tables

1.1 Supplementary Figures

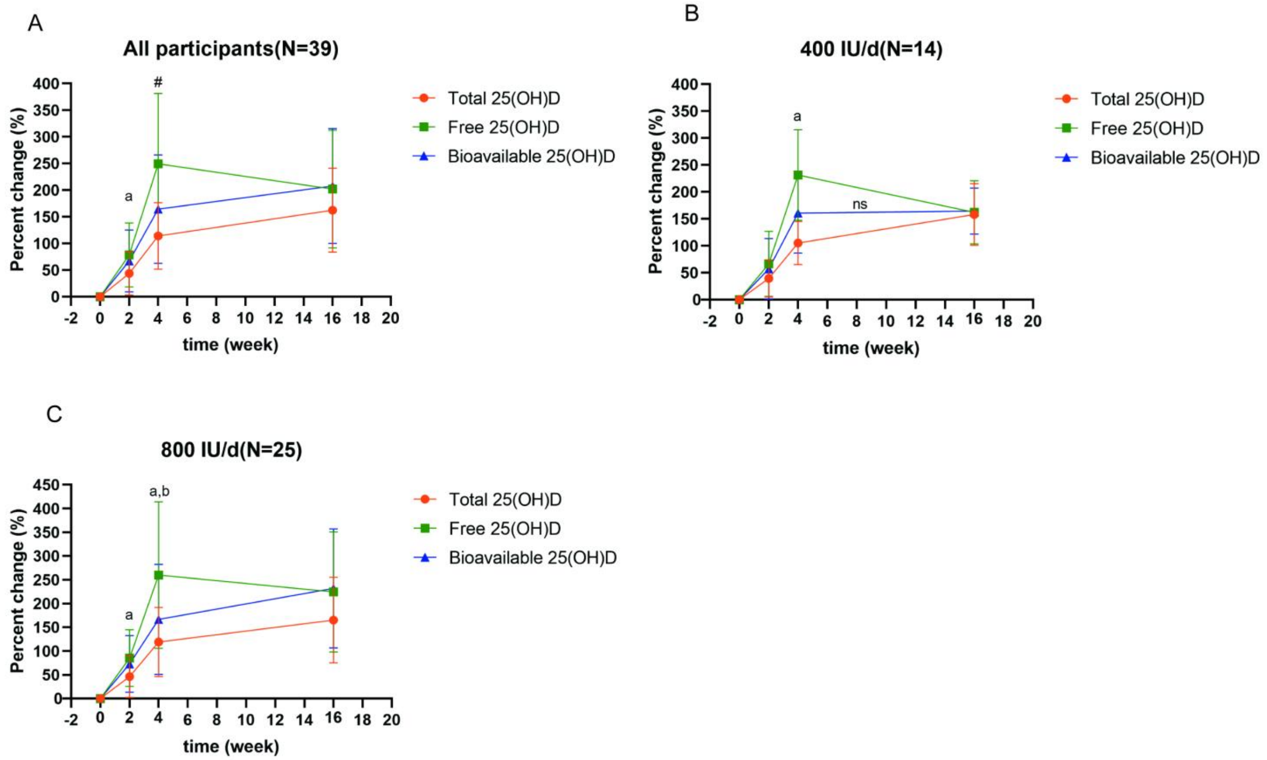

Supplementary Figure 1. Total-25(OH)D, Free-25(OH)D, and Bio-25(OH)D response to vitamin D supplementation. #, compared with other two indicators; a, Free-25(OH)D vs. Total-25(OH)D; b, Free-25(OH)D vs. Bio-25(OH)D; ns, no difference between week 4 and week 16 for Bio-25(OH)D. Repeated measures analysis of variance was used to compare means between the groups of interest. *P*<0.05 was considered statistically significant.
